# The unmet needs in suicide prevention among nursing staff

**DOI:** 10.1192/j.eurpsy.2025.2359

**Published:** 2025-08-26

**Authors:** A. Litta, A. Nannavecchia, V. Favia, C. Cutrone, F. Bafaro

**Affiliations:** 1 University of Bari “Aldo Moro”; 2 AReSS Puglia- Regional Strategic Agency for Health and Social; 3 Istituto Tumori Giovanni Paolo II, IRCCS, National Cancer Institute; 4 University of Bari Aldo Moro, Bari; 5 Healthcare Residence Villa Iris, Mesagne, Italy

## Abstract

**Introduction:**

Nurses can play a critical role in suicide prevention, especially in hospital settings. The management of patients at risk of suicide requires specific nursing skills, including early recognition of warning signs and knowledge of preventive and protective strategies. Knowing risk factors and recognizing warning signs for suicide should be part of the professional background of any nurse training programs.

**Objectives:**

In this study we aimed to investigate nurses’s comfort, confidence and competence related to preventing suicide with a view to identify correct preventive strategies regarding the nursing management of patients at risk of suicide.

**Methods:**

Our study presents the preliminary descriptive findings from an online survey in nursing staff working in different areas (medical, surgical, critical and emergency) in the Puglia Region. The survey aimed to assess the current knowledge, attitudes, behaviors and training needs of nurses regarding suicide prevention. 84 nurses working in the Puglia Region filled out the questionnaire.

**Results:**

Data highlighted that the majority of participants (81%) recognized the significant role of the nurses in the management of a patient at risk for suicide but only 14.3% believed they had specific training on patients at risk of suicide. 50% of them stated they do not have adequate preparation regarding the possible preventive strategies in suicidal patient. 57.1% of interviewed reported that they had never become aware of protocols or guidelines on the prevention and management of hospitalized patients at risk of suicide. Only 14.3% of those interviewed are satisfied with adequate training on suicidal risk factors in hospitalized patients and even only 7.1% state that they have never received adequate training on possible nursing interventions for the prevention of suicidal risk. Although endorsed suicide prevention guidelines in the health institution where they work, 57.1% responded that they have never viewed them and only 38.9% partially.

**Conclusions:**

Our study highlighted the need to implement specific training programs for nurses on the management of patients at risk of suicide.

**Disclosure of Interest:**

None Declared

